# Antimicrobial resistance of enteric pathogens in the Military Health System, 2009 – 2019

**DOI:** 10.1186/s12889-022-14466-1

**Published:** 2022-12-08

**Authors:** Hayley Ashbaugh, Connor D. Pomeroy, Mona Baishya, Kathleen Creppage, Sara Bazaco, Myles Johnson, Kenji Matsumoto, Upendra Bhattarai, Nicholas Seliga, Paul Graf, Uzo Chukwuma

**Affiliations:** 1 Walter Reed Army Institute of Research, U.S. Army Medical Research Directorate-Georgia, 99 Kakheti Highway, 0198 Tbilisi, Georgia; 2 Oak Ridge Institute for Science and Education, Defense Centers for Public Health - Portsmouth, 620 John Paul Jones Circle, Suite 1100, Portsmouth, VA 23704 USA; 3 Public Health Directorate, Armed Forces Health Surveillance Division, Global Emerging Infections Surveillance Branch, 11800 Tech Road, Silver Spring, MD 20904 USA; 4 Deloitte Consulting LLP, 1919 N Lynn St Suite 1500, Rosslyn, VA 22209 USA; 5 Defense Centers for Public Health - Portsmouth, 620 John Paul Jones Circle, Suite 1100, Portsmouth, VA 23704 USA; 6 Naval Health Research Center, San Diego, CA USA; 7 Present Address: Naval Medical Research Unit No. 6, Lima, Peru; 8 Henry M. Jackson Foundation for the Advancement of Military Medicine, Inc., Defense Centers for Public Health - Portsmouth, Portsmouth, United States; 9 ManTech International Corporation, Defense Centers for Public Health - Portsmouth, Portsmouth, United States

**Keywords:** Enteric pathogens, Antimicrobial resistance, Infectious disease surveillance, *Salmonella*, *Shigella*, *Campylobacter*, *Escherichia coli*

## Abstract

**Background:**

Acute diarrhea (AD) can have significant impacts on military troop readiness. Medical providers must understand current trends of enteropathogen antimicrobial resistance (AMR) in service members (SMs) to inform proper, timely treatment options. However, little is known of enteric pathogen profiles across the Military Health System (MHS). The primary objectives of this study were to identify gaps in enteric pathogen surveillance within the MHS, describe the epidemiology of AMR in enteric pathogens, and identify trends across the MHS both within the Continental United States (CONUS) and outside of the Continental United States (OCONUS).

**Methods:**

Health Level 7 (HL7)-formatted laboratory data were queried for all specimens where *Salmonella*, *Shigella*, and *Campylobacter* species, as well as Shiga toxin-producing *Escherichia coli* (*E. coli*) (STEC) were isolated and certified between 1 January 2009 - 31 December 2019. Antibiotic susceptibility testing (AST) results were queried and summarized where available. Descriptive statistics were calculated for each organism by specimen source, year, and susceptibility testing availability.

**Results:**

Among a total of 13,852 enteric bacterial isolates, 11,877 (86%) were submitted from CONUS locations. Out of 1479 *Shigella* spp. and 6755 *Salmonella* spp. isolates, 1221 (83%) and 5019 (74%), respectively, reported any susceptibility results through the MHS. Overall, only 15% of STEC and 4% of *Campylobacter* spp. specimens had AST results available. Comparing AST reporting at CONUS versus OCONUS locations, AST was reported for 1175 (83%) and 46 (78%) of *Shigella* isolates at CONUS and OCONUS locations, respectively, and for 4591 (76%) and 428 (63%) of *Salmonella* isolates at CONUS and OCONUS locations, respectively.

**Conclusions:**

This study revealed inconsistent enteropathogen AST conducted across the MHS, with differing trends between CONUS and OCONUS locations. Additional work is needed to assess pathogen-specific gaps in testing and reporting to develop optimal surveillance that supports the health of the force.

**Supplementary Information:**

The online version contains supplementary material available at 10.1186/s12889-022-14466-1.

## Introduction and background

Acute diarrhea (AD) continues to be a leading cause of morbidity in the United States, with an estimated 179 million cases annually resulting in 1.5 million outpatient visits, 200,000 hospitalizations, and 300 deaths [1, 2]. AD is defined as three or more loose or watery stools per day, for a duration lasting 14 days or fewer [3], although other gastrointestinal signs and symptoms may be present. Viral pathogens (particularly norovirus) acquired through the consumption of contaminated food items or environmental sources are the most common causes of acute diarrheal illness among U.S. military trainees and in the U.S. overall [2, 4], but bacterial pathogens have been more frequently associated with diarrheal infections and outbreaks among deployed personnel or traveler populations [5]. Although AD is often self-limiting, with mild symptoms commonly resolving within 5 days, in moderate to severe cases of bacterial AD, antibiotics such as macrolides (e.g., azithromycin) and fluoroquinolones (e.g., ciprofloxacin) are recommended for empiric management [6, 7]. Antibiotic therapy has been shown to reduce the severity of symptoms and to shorten the duration of illness [8]. Timely resolution of clinical illness is important, since prolonged duration has been associated with chronic post-infectious sequelae [9]. However, over the past two decades, bacterial AD pathogens have shown increasing resistance to these first-line antibiotics, and this has been associated with negative clinical outcomes such as treatment failure and delayed resolution of clinical illness [9–12].

This emergence of antimicrobial resistance (AMR) has been linked to several factors including varying prescription practices of healthcare professionals (in some cases over prescription of antibiotics), inconsistent patient medication adherence, self-medication, travel abroad, and non-human use of antimicrobials [8, 13]. The regular use of antimicrobial agents in food animals has generated resistance in commensal bacteria, which presents a potential source for human acquisition of resistance from the consumption of food producing animals [14]. Due to these external pressures, rates of AMR in diarrheal pathogens have been increasing [12].

This recent increase of AMR in enteropathogens is of concern to U.S. service members (SM). SMs are at increased risk of developing AD due to their deployments to overseas locations [15], and exacerbated by the fact that a number of those locations have concerning levels of AMR in relevant pathogens [9–12]. Episodes of diarrheal disease can have detrimental impact on troop readiness and mission operations, underscoring the importance of timely resolution of symptoms in this population [16–19]. It is therefore essential for military medical providers to understand the current trends in resistance profiles of enteropathogens in SMs to inform proper, timely treatment options.

However, across the Military Health System (MHS), very little is currently known regarding AD bacterial pathogen AMR profiles among SMs. Therefore, the primary objectives of this paper are to describe the epidemiology of AMR in enteric pathogens and identify trends across the MHS in both the Continental United States (CONUS) and outside of the Continental United States (OCONUS).

## Methods

### Identification of enteric pathogen samples

Health Level 7 (HL7)-formatted laboratory microbiology and chemistry data (including molecular tests and antigen tests) from Composite Health Care System (CHCS) data were used to identify positive test specimens of interest for all military beneficiaries who received care within the MHS from January 1 2009 through December 31 2019. While the intent of this study is to highlight potential implications to SMs, beneficiaries  0-17 years of age were included in the analysis, as individuals in this age range are dependents who often receive care within the MHS. Additionally, since these children often live in the same household as the SM parent, they are likely exposed to the same food-borne pathogens. Methods used for extracting and aggregating microbiology data from electronic health records have been previously published [20].

The specimens of interest included important diarrheagenic bacteria with potential for development of AMR: *Campylobacter* species (spp.), *Escherichia coli (E. coli)*, *Salmonella* species, and *Shigella* species. Laboratory data were queried using search terms listed in Supplementary Table 1 for each specimen. *E. coli* isolates were limited to a single pathotype (Shiga toxin-producing *E. coli* ((STEC)) that can result in severe (and often bloody) diarrhea.

The following search terms or a combination were used to identify sample type records of isolates: ‘stool’, ‘fecal’, ‘rectum’, ‘rectal contents’, ‘feces’, ‘rectal swab’, or if the ordered test included any combination of the following terms: ‘fecal’, ‘feces’, ‘stool culture’, ‘GI panel’, ‘gastroent’, ‘E.coli – enterohemorrhagic’.

A unique case was identified as a single positive pathogen of interest identified per person, with a minimum interval of 14 days between infections of the same organism of interest for any patient. For example, if *Campylobacter* was detected on Day 1, a second detection of *Campylobacter* on Day 10 would not be counted for the same patient, but on Day 16, a detection of *Campylobacter* would be counted as an additional infection. Conversely, a detection of *Shigella* on Day 10 would be counted as a separate infection in the same patient. Under this case definition (‘one isolate per patient per 14 days’), an individual could be counted more than once if they a) tested positive for different pathogens of interest or b) tested positive for the same pathogen after the 14 day minimum interval.

Based on these criteria, 223 participants had detections of two unique organism of interest, two participants had detections of three, and one participant had detections of all four organisms of interest. The majority (*n* = 13,021, 98%) of patients had only one organism of interest detected. When repeated infections with the same organism are considered, there were 464 participants with two repeated organisms of interest detected, 42 participants with three, and 12 patients with four. Two participants had only a single organism of interest detected, yet they experienced infection seven times. While we cannot state whether this detection of the same pathogen indicates reinfection, as collection of subtyping information was beyond the scope of this work, it is important to note that a small number of participants were observed to have a high number of repeated detections of the same organism.

In the case of multiple positive results from the same patient, positive lab findings from the microbiology results were prioritized over chemistry to capture antibiotic susceptibility testing (AST), which is only available in microbiology HL7 data. As a result, only one sample (the one from microbiology) would be included in the case of both microbiology and chemistry results being available.

### Antibiotic resistant testing results

BacLINK and WHONET [21] software packages were used to re-format the microbiology data and its AST results into a usable output for analysis. While the number of susceptible organisms was provided for all antibiotic-organism combinations, antibiotic susceptibility percentages where fewer than 30 isolates were tested for a given organism in a given year were suppressed, as such results would not be recommended for inclusion in AST for clinical use since the results would be considered statistically unstable [22]. Analysis of susceptibility results were limited to the first isolate per person per specimen.

### Antibiotic susceptibility testing and laboratory testing methods

Specimen identification and AST were performed primarily by automated methods to media type and manufacturer. All identification was performed primarily by automated methods cleared by the U.S. Food and Drug Administration. Most laboratories used Vitek 2 (Biomerieux), while MicroScan (Beckman Coulter) was used by the remaining laboratories except for two that used Phoenix (BD) and one that used Sensititre (Thermofisher) for AST. Matrix-associated laser desorption/ionization-time of flight (MALDI-TOF) mass spectrometry on BioTyper (Bruker) or Vitek MS (Biomerieux) was used for bacterial identification by a small minority of laboratories. Some laboratories also used manual or semiautomated methods for identification such as API (Biomerieux), and AST such as Kirby-Bauer disk diffusion, and gradient strip diffusion (Etest, Biomerieux) as primary or secondary methods, especially for *Campylobacter* species. All military treatment facility (MTF) laboratories are accredited by the College of American Pathologists (CAP) and must meet strict performance criteria and pass regular proficiency testing to maintain accreditation regardless of testing methodology. Determining whether laboratories used FDA breakpoints or Clinical Laboratory Standards Institute (CLSI) breakpoints (which were updated numerous times during this evaluation period) was beyond the scope of this study. Disk diffusion zone sizes are not reported in HL7 data, and minimal inhibitory concentrations (MICs), when reported, are limited to the range on the AST panel.

### Geographic stratification

Samples were taken from numerous MTFs globally. To examine differences by geography, MTFs were categorized based on geographic location: CONUS (continental United States - including Alaska and Hawaii) and OCONUS (outside of the continental United States). Results were also stratified based on Geographic Combatant Command (GCC), which are geographic areas of responsibility for the U.S. military [23]. There are 7 main GCCs, each of which are responsible for military operations and troop readiness in their assigned regions: SOUTHCOM (all countries south of Mexico excluding Antarctica, plus the Caribbean Islands except for U.S. Virgin Islands, Puerto Rico and Bahamas), AFRICOM (African continent except for Egypt), EUCOM (European countries including the Asian parts of Russia and Turkey, plus Israel), NORTHCOM (extending north to south from Canada to Mexico, including U.S. Virgin Islands, Puerto Rico and Bahamas), INDOPACOM (Asia-Pacific region south of Russia – extending west to east from India to Mongolia, and all Pacific island countries), CENTCOM (Middle East, Central and South Asia plus Egypt, excluding Israel and Turkey), and SPACECOM (space, no data available for analysis).

### Data analysis

Descriptive statistics were calculated using SAS software, V. 9.4 across years and genera. Frequencies and percentages were reported. This project was reviewed by a NMCPHC Exemption Determination Official (EDO) to determine if review and approval by the servicing institutional review board (IRB) would be required prior to commencement of research activities. Based on the project purpose and objectives, this study is considered a research activity involving human subjects; however, the study is exempted from IRB review process because it a public health surveillance activity.

## Results

### Patient demographic characteristics

There were 13,852 specimens that contained *Campylobacter* spp., STEC, *Salmonella* spp., or *Shigella* spp. among 13,247 MHS beneficiaries between the calendar years 2009 and 2019. Tables 1 and 2 are deduplicated to show all MHS beneficiaries with at least one pathogen of interest over the entire time frame. Most beneficiaries were male (*n* = 7403, 56%), between the ages of 18-45 years old (*n* = 5956, 45%), and from CONUS locations (*n* = 11,350, 86%). More than half (*n* = 7587, 57%) of the 13,247 beneficiaries were dependents. Army SMs had the highest number of beneficiaries with a positive specimen (*n* = 5891, 44%).

**Table 1:** Demographics of MHS Beneficiaries with at least one Enteric Bacterial Isolate, MHS, 2009-2019 (*n* = 13,247)

	Frequency (n)	Percent (%)^**a**^
**Sex**		
Male	7403	55.9
Female	5844	44.1
**Age Group (yrs)**		
0-4	3196	24.1
5-17	1590	12.0
18-45	5956	45.0
45+	2505	18.9
**Beneficiary Category**		
ActiveDuty	3581	27.0
Dependent	7587	57.3
Recruit	209	1.6
Retired	1104	8.3
Other	766	5.8
**Patient Service**		
Air Force	3175	24.0
Army	5891	44.4
Marines	1229	9.3
Navy	2439	18.4
Other	513	3.9
**Geography**		
CONUS	11,350	85.7
OCONUS	1897	14.3
**Sub-Regional Geography**		
US WEST	2732	20.6
US MIDWEST	476	3.6
US NORTHEAST	139	1.1
US SOUTH	2694	20.3
US SOUTH ATLANTIC	5309	40.1
OCONUS	1897	14.3
**Total**	13,247	

Data source: HL7-formatted microbiology and chemistry databases

*CONUS* Continental United States

*OCONUS* Outside Continental United States

^a^Per cent is calculated as the number of patients in each category divided by the total number of MHS beneficiaries in that region with an enteric pathogen

**Table 2:** Demographics of MHS Beneficiaries with at least one Enteric Bacterial Isolate, by CONUS/OCONUS, MHS, 2009-2019 (*n* = 13,247)

	CONUS		OCONUS	
	Frequency	Percent^**a**^	Frequency	Percent^**a**^
**Sex**				
Male	6227	54.86	1176	62.0
Female	5123	45.14	721	38.0
**Age Group (yrs)**				
0-4	3016	26.6	180	9.5
5-17	1423	12.5	167	8.8
18-45	4649	41.0	1307	68.9
45+	2262	19.9	243	12.8
**Beneficiary Category**				
Active Duty	2639	23.2	942	49.7
Dependent	6949	61.2	638	33.6
Retired	1010	8.9	94	5.0
Recruit	199	1.8	10	0.5
Other	553	4.9	213	11.2
**Patient Service**				
Air Force	2457	21.6	718	37.8
Army	5316	46.8	575	30.3
Marines	1142	10.1	87	4.6
Navy	2096	18.5	343	18.1
Other	339	3.0	174	9.2
**Total**	11,350		1897	

Data source: Health Level 7 (HL7)-formatted microbiology and chemistry databases

Prepared by the EpiData Center, NMCPHC, on 17 Aug 2022

^a^Per cent is calculated as the number of patients in a given category divided by the total number of MHS beneficiaries in that region with an enteric pathogen

There was a greater proportion of male versus female participants in both CONUS and OCONUS locations, and age groups displayed different patterns CONUS versus OCONUS, with 27% of CONUS beneficiaries falling into the 0-4 years age group versus 10% of OCONUS beneficiaries falling into this group. However, the 18-45 year age group was the largest age group for both CONUS and OCONUS locations. Most of the beneficiaries from CONUS locations with a positive specimen were dependents (61%) and affiliated with the Army (47%); however, the majority of beneficiaries from OCONUS locations were active duty (50%) and affiliated with the Air Force (38%).

### Specimen characteristics

Of the 13,852, identified organisms (12,174 from culture records and 1678 from nonculture (chemistry) records), the most frequent target organism was *Salmonella* (*n* = 6755, 49%), and the least frequent was STEC (*n* = 691, 5%) (Table 3). As expected, most organisms (*n* = 12,574, 91%) were detected in stool samples. Among organisms detected in stool samples, *Salmonella* spp. accounted for nearly half (*n* = 5823, 46%), followed by *Campylobacter* spp. (*n* = 4819, 38%), *Shigella* spp. (*n* = 1322, 11%) and STEC (*n* = 610, 5%). Most of the bacteria isolated from non-stool samples were detected in urine. *Salmonella* spp. made up nearly three-fourths of the organisms isolated from non-stool samples (*n* = 932, 73%), followed by *Shigella* spp. (*n* = 157, 12%).

**Table 3:** Frequency of Isolated Organisms by Stool/Non-Stool, MHS, 2009 – 2019 (*n* = 13,852)

	Stool		Non-Stool		Total Samples Detected	Percent of All Samples Detected
	No Detected	Percent of Stool Samples Detected^a^	No. Detected	Percent of Non-Stool Samples Detected^a^		
**Bacterial Genus**						
Shiga toxin-producing *E. coli*	610	4.9	81	6.3	691	5.0
*Campylobacter*	4819	38.3	108	8.5	4927	35.5
*Salmonella*	5823	46.3	932	72.9	6755	48.8
*Shigella*	1322	10.5	157	12.3	1479	10.7
Total	12,574	90.8^b^	1278	9.2^b^	13,852	100.0

Data source: HL7-formatted microbiology and chemistry databases

^a^Per cent is based off the total number of organisms that fell into a respective category

^b^Per cent is based off the total number of organisms detected

**Fig. 1:**
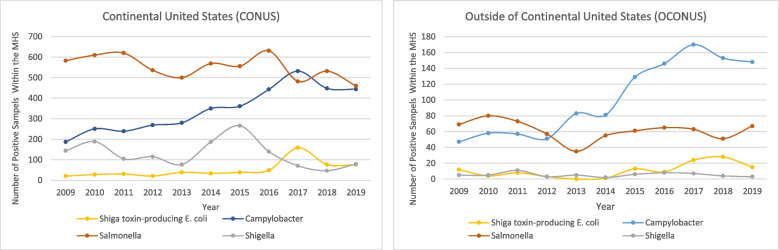
Frequency of Selected Enteric Pathogens within the MHS by Laboratory Certification Year, CONUS/OCONUS, 2009-2019 (*n* = 13,852). Data source: HL7- formatted laboratory CHCS data

Approximately 86% (*n* = 11,877) of all organisms were identified in CONUS locations. With the exception of 2017, *Salmonella* spp. was the most frequently isolated enteric bacteria in CONUS locations, and except for 2009-2012, *Campylobacter* spp. was the most frequently isolated enteric bacteria in OCONUS locations (Fig. 1). The increase in STEC infections in CONUS during 2017 was due to a large outbreak of STEC O157 among 244 Service members at Marine Corps Recruit Depot (MCRD) San Diego [24]. While the total number of 244 includes all confirmed, probable, and suspect cases, CHCS data includes only confirmed and probable cases, with “confirmed” defined as culture positive with pulsed field gel electrophoresis (PFGE) performed by the California State Public Health Lab, and “probable” defined as STEC antigen positive via testing at Naval Medical Center San Diego (NMCSD), but either culture negative or not performed/pending. Within CHCS data, 87 cases were reported from the MCRD clinic and 7 cases were reported by NMCSD from the 2017 outbreak.

When stratified by GCC, most organisms came from specimens that were collected from NORTHCOM locations (*n* = 11,877, 86%), followed by EUCOM (*n* = 1199, 9%), INDOPACOM (*n* = 733, 5%), CENTCOM (*n* = 32, 0.2%) and SOUTHCOM (*n* = 11, 0.1%). No samples were collected from AFRICOM. With the exception of Camp Lemonnier, Djibouti, the majority of service members in AFRICOM operate in small groups and access to medical care can be limited [25]. There were no *Campylobacter* spp., STEC, or *Shigella* spp. identified from CENTCOM. In INDOPACOM, a half (50%) of detected organisms were *Campylobacter* spp. and 46% were *Salmonella* spp. In NORTHCOM, over half of isolates were *Salmonella* spp. In EUCOM, 63% were *Campylobacter* spp. (Supplementary Table 2).

### Antibiotic susceptibility testing

Among all enteric pathogens of interest identified within the MHS, 6524 (47%) had AST results for at least one antibiotic (Supplementary Table 3). Susceptibility testing was performed more frequently for isolates from stool sources than non-stool and from CONUS versus OCONUS locations. Specimens containing *Shigella* or *Salmonella* spp. frequently had susceptibility results (83% of *Shigella* spp. specimens, 74% of *Salmonella* spp. specimens) compared to specimens that contained STEC or *Campylobacter* spp. (15% of all STEC specimens and 4% of all *Campylobacter* spp. specimens). Out of the 6524 specimens that had AST results, 5995 (92%) came from CONUS locations. Most of these were stool specimens (*n* = 5270). The majority of these 5995 CONUS specimens with AST results contained *Salmonella* spp. (*n* = 4591, 76%). Of the 529 samples that had AST results that came from OCONUS locations, the majority were stool (*n* = 431, 82%) and contained *Salmonella* spp. (*n* = 359). For both CONUS and OCONUS locations, specimens containing STEC were the least frequent**.** Out of all MTFs that reported AST results (*n* = 308) for enteric pathogens of interest, 50 (16%) reported susceptibility results for STEC or *Campylobacter* spp.

Of the specimens that had AST results, trimethoprim/sulfamethoxazole (*n* = 6204, 95%), ampicillin (*n* = 6161, 94%) and ciprofloxacin (*n* = 4978, 76%) were the most frequently tested antibiotics, while the third generation cephalosporin ceftizoxime (*n* = 2, 0.03%) was the least frequently tested among a number of other antibiotics infrequently included in AST (Supplementary Table 4).

Supplementary Table 5 shows the susceptibility results of all tested organisms and the per cent susceptible for those organisms that had at least thirty total isolates tested in a given year, indicating that the estimates are considered statistically stable. Most *Salmonella* or *Shigella* spp. specimens that underwent AST had results for aminopenicillins, quinolones or sulfonamides. For each antibiotic tested, at least 90% of *Salmonella* spp. isolates from CONUS locations were susceptible. More resistance was seen when evaluating *Shigella* spp. isolates, with ampicillin (43-85%), fluoroquinolones (88-100%), and trimethoprim-sulfamethoxazole (29-61%) showing reduced levels of susceptibility**.**

*Campylobacter* spp. and STEC do not have any susceptibility results reported for all years due to infrequent AST. Supplementary Table 6a (*Campylobacter* spp.) and 6b (STEC) show the number of specimens that had AST testing for these two species**.**

## Discussion

### Pathogen detection

Between Jan 1, 2009 and December 31, 2019, 13,852 specimens containing an enteric pathogen of interest were identified in 13,247 patients from the MHS HL-7 Microbiology and Chemistry laboratory datasets. However, due to the low proportion of samples with available AST results, the AMR patterns of enteric bacteria during this time period were difficult to describe.

The populations with samples containing enteric pathogens of interest differed between CONUS and OCONUS locations, with a higher proportion of those within CONUS being either 0-4 years or 18-45 years of age, while those OCONUS were mostly 18-45 years of age. This likely reflects the underlying distribution of the military personnel stationed in each area – there are fewer children OCONUS than CONUS, and the majority of personnel OCONUS were active duty service members [26]. This is also reflected in the differences by beneficiary category across these regions. The higher overall frequency of samples from NORTHCOM is likely due to a greater number of people being stationed in the Area of Responsibility (AOR), rather than being related to additional risk for that population. Unfortunately, due to a lack of population denominators for each GCC, we were unable to provide direct comparisons.

The enteric pathogens identified in these samples varied by region, as well as by GCC. Overall, in samples from CONUS locations, *Salmonella* spp. were most frequently detected, while *Campylobacter* spp. were most frequently detected in samples from OCONUS. However, there is a noticeable change in frequency for *Campylobacter* spp. from 2013 to 2018 in CONUS locations, with the number of *Campylobacter* samples increasing to become approximately equal to *Salmonella* in 2017-2018. The recent similarity in frequency between *Salmonella* and *Campylobacter* mirrors what was seen in the general US population as captured in FoodNet [27]. However, in that database *Salmonella* and *Campylobacter* have been detected at similar frequencies since 1999. The earlier differences between our MHS population and the general US population may have been due to limited capacity to detect *Campylobacter* at the MTFs, as discussed in additional detail below.

The differences in most frequently detected pathogen in CONUS versus OCONUS locations likely reflect a difference of exposure, as the *Campylobacter* predominance over *Salmonella* in the OCONUS region is primarily driven by the results from INDOPACOM and EUCOM. The high risk of diarrhea due to *Campylobacter* infection within Asia is well known [28, 29], and so *Campylobacter* spp. comprising nearly half of enteric isolates from INDOPACOM is unsurprising. In the EUCOM AOR, since 2006, the European Union has enacted regulations to control salmonellosis in their poultry production systems – this includes regular testing, culling, strict biosecurity, and vaccination programs [30]– which has significantly reduced population exposure to *Salmonella* in the region. Unfortunately, however, information on potential exposure to local animal products, particularly OCONUS, is limited.

GCC differences were even more pronounced, especially those in CENTCOM where only S*almonella* was reported. This could have been due to a lack of reporting rather than indicative of *Salmonella* being the only bacterial etiology of AD in CENTCOM during this time period; other work has identified multiple bacterial etiologies of AD among SMs in this GCC [19]. Also surprising was that SOUTHCOM presented no STEC samples and very few *Shigella* and *Campylobacter* cases – however their numbers overall were quite low. These differences might reflect variations in sample collection practices or capabilities in deployed settings [19, 31], or may be due to a reluctance of patients to provide stool samples.

### Antimicrobial susceptibility testing reporting

Generally, there were fewer AST results than expected, with only 47% of isolates associated with an AST report. Laboratory testing practices (specifically culturing) can differ by provider, facility, and geographic region, and this extends to antimicrobial resistance testing. The number of fecal isolates subjected to AST is likely influenced by numerous factors in the clinical laboratory, including staffing availability, monetary concerns, and the availability of treatment and laboratory reference guidelines.

Particularly concerning is the low level of AST for *Campylobacter* spp. (4% of isolates) given the frequency with which it was detected. CLSI guidance for *Campylobacter* - covering only *C. jejuni* and *C. coli* against ciprofloxacin, erythromycin (a proxy for azithromycin) and tetracycline - indicates that susceptibility testing should be considered only for epidemiology purposes or in cases of prolonged or severe infections, which could explain the paucity of AST data available in our datasets [32]. However, given that mild-moderate AD is often self-resolving, yet patients in our study were presenting for medical care, we might expect that a higher proportion of *Campylobacter* spp. isolates would have been from cases experiencing prolonged or severe infections for which AST would have been indicated. Another potential reason for low AST could be due to the difficulty of testing fastidious *Campylobacter*, and with the lack of disc diffusion breakpoints until 2016, many clinical laboratories within the MHS have chosen not to perform AST on this organism. Rates of testing were similarly low for STEC (15%) which is unsurprising given the recommendation to avoid antibiotic treatment for infected patients due to concerns of increased risk of Hemolytic Uremic Syndrome [7, 33]. Of 308 MTFs reporting AST results on isolates included in our study, only 50 MTFs reported an AST for either *Campylobacter* or STEC, which indicates that tests are being done infrequently across the MHS.

The CLSI recommends AST on all *Shigella* spp. isolates against ampicillin, fluoroquinolones, and trimethoprim/sulfamethoxazole. As only 83% of *Shigella* isolates underwent susceptibility testing within the MHS, there is room for improvement for AST compliance, but it had the highest testing percentage of all the pathogens we examined.

Unfortunately, the MHS data systems do not provide for species/serovar level ICD codes, but it is likely that the majority of the Salmonella pathogens detected were *S. enterica.* According to the CLSI, all isolates of typhoidal *Salmonella* should be tested routinely, however, testing is not indicated for intestinal non-typhoidal *Salmonella* [34]. If MTF laboratories were following these guidelines, the amount of testing for *Salmonella* would, therefore, likely be close to zero. However, without further specificity it is difficult to make a true comment on AST compliance for Salmonella.

### Antimicrobial susceptibility testing trends

The observed high susceptibility rates seen in *Salmonella* isolates from CONUS locations very closely match rates from national enteric bacteria surveillance reported by the National Antimicrobial Resistance Monitoring System (NARMS) [35]. However, when > 30 isolates were available in the MHS data to show per cent susceptible at OCONUS locations, *Salmonella* isolates consistently showed decreased susceptibility to both ampicillin and trimethoprim-sulfamethoxazole at OCONUS versus CONUS locations. Of note, it is unclear which breakpoints, FDA vs. CLSI, were used for ciprofloxacin and levofloxacin against *Salmonella* spp. (MHS data only), which could potentially make the susceptibility results difficult to interpret. The CLSI MIC breakpoints for ciprofloxacin decreased substantially (and disk diffusion zone size breakpoints correspondingly increased) for *Salmonella Typhi* and extraintestinal isolates of *Salmonella* spp. in 2012 and extended to all *Salmonella* spp. from all sources in 2013. The FDA breakpoint changes trailed those of the CLSI by several years, but depending on the automated AST system and panel used, may not have been implemented consistently throughout the MHS. Assuming that the updated CLSI breakpoints were implemented during the course of this study, if anything, would cause an increase in the percent of nonsusceptible isolates in later years vs. earlier years. As ciprofloxacin susceptibility was consistently > 90% during each year, it does not appear that the change in breakpoints affected the overall susceptibility rate for ciprofloxacin against *Salmonella* spp. (Supplementary Table 4). Likewise, the CLSI breakpoints for ciprofloxacin and levofloxacin were changed for all Enterobacteriales (excluding *Salmonella* spp. but including *Shigella* spp. and STEC) in 2019. It does not appear that the change in the last year of this study had any effect on *Shigella* spp. or STEC susceptibility rates; a slight trend towards a decrease in ciprofloxacin and levofloxacin susceptibility for *Shigella* spp. predated the CLSI breakpoints change (Supplementary Table 4) while no STEC isolates underwent AST for either fluoroquinolone in 2019 (Supplementary Table 5).

Due to the lab analysis being completed at multiple sites and with multiple analyzers and methods (all potentially with different FDA approved versions used on their analyzers) the only way to standardize the data was to use their Susceptible, Intermediate, or Resistant breakpoint interpretations. MIC values are not always reported in the patient record and therefore not consistently traceable. The breakpoint interpretations represented the CLSI or FDA version that the local lab has validated for their individual method and is therefore applicable as a standard way to report antibiotic sensitivity.

*Shigella* spp. AST patterns of susceptibility to ampicillin closely mirror those seen in NARMS except for 2015 and 2017, where the MHS results differed from those reported in NARMS. In 2015, NARMS reported 43% of *Shigella* isolates resistant to ampicillin, while MHS data showed 15% of *Shigella* isolates as non-susceptible; in 2017, NARMS reported that 45% of *Shigella* isolates were resistant to ampicillin, while MHS data showed only 57% of *Shigella* isolates as non-susceptible to ampicillin. Susceptibility of *Shigella* to trimethoprim-sulfamethoxazole in the MHS also reflected the increase in susceptibility seen in NARMS data from 2011 to 2012 (from 67% resistant to 43% resistant as reported by NARMS and from 62% non-susceptible to 39% non-susceptible as reported from MHS CONUS data, for the years 2011 and 2012, respectively), but the MHS CONUS data showed a sharp reduction in susceptibility in 2014 (60% non-susceptible) while NARMS did not, reporting 41% of Shigella isolates tested resistant to trimethoprim-sulfamethoxazole. Both NARMS and MHS CONUS data show an increase in *Shigella* spp. resistance and non-susceptibility to ciprofloxacin from 2015 to 2019, with NARMS showing resistance increases from 3% in 2015 to 18% (preliminary) in 2019, and MHS CONUS data showing increases in non-susceptibility from 2% in 2015 to 11% in 2019 (no susceptibility data available for 2018).

There were too few AST results reported for *Campylobacter* and STEC to make any meaningful comparisons to external sources.

### Study limitations and strengths

There are some inherent limitations to the work presented, including data sources, types of surveillance systems, and testing limitations.

The HL7 data generated within the CHCS that is included in the laboratory microbiology and chemistry datasets are collected from fixed military MTFs. These data do not include records from shipboard facilities, battalion aid stations, purchased care (in civilian clinics and hospitals outside of the MHS), or in-theater facilities. This may result in a severe underestimation of the burden of enteric bacterial organisms in specific geographic regions where beneficiaries choose, prefer, or have no alternative other than to seek care outside of the MHS. The MHS system has no visibility on pathogen detection or AMR patterns for such individuals. This leaves a significant gap in antimicrobial resistance surveillance for enteric pathogens within the MHS, and particularly in CENTCOM, the low isolate numbers reported may have been due to pathogen and testing data captured in the Theater Medical Data Store which is not accessible through CHCS.

AST performed by clinical laboratories, such as in MTFs, is designed for patient management, and this directs when to perform AST and which antibiotics to include; whereas surveillance programs often perform AST on additional antibiotics beyond those typically used in patient management. There are, therefore, limitations to applying data from laboratories within the MHS to answer surveillance questions since MTFs are focused on individual patient management rather than surveillance. For instance, except for *Camplyobacter coli* and *jejuni* and *Salmonella Typhi*, there are no breakpoints for macrolide antibiotics, likely explaining the absence of azithromycin AST data for *Salmonella* spp., *Shigella* spp., and STEC in this dataset. Additionally, many MTFs report superfluous AST results for many antibiotics that are not recommended for reporting such as aminoglycosides, 1st and 2nd generation cephalosporins against *Salmonella* spp. and *Shigella* spp. (Supplementary Table 3). To get a clearer, more complete picture of resistance factors within enteric pathogens seen in SMs, targeted surveillance activities should continue in order to complement or supplement public health information gained from existing clinical testing.

Information on resistance that could be acquired by SMs and beneficiaries through exposure to animal products, particularly OCONUS, is lacking. While the role of the MTF is to conduct clinical testing, there is a need for AMR surveillance for antimicrobial classes that are of human clinical importance, since pathogens associated with animal-source food products can carry AMR genes resulting in these resistant phenotypes. For example, globally, antimicrobials of human clinical importance are sometimes used in livestock, which may enhance selection for resistance to fluoroquinolones and macrolides, which could then potentially be transferred into the human population [14].

As culture independent diagnostic testing steadily continues to replace traditional bacterial culture, fewer isolates will be available on which to perform antimicrobial susceptibility testing. Since susceptibility testing is not an option when pathogen detection is performed only via molecular methods, future efforts to surveil AMR threats in these populations may need to consider newer technologies such as metagenomics and other sequencing techniques. However, without the isolate no phenotypic results will be available, which is concerning, as those results are potentially more meaningful and useful.

## Conclusion

Despite these limitations, to the authors’ knowledge, this is the first study examining antibiotic resistance patterns of enteric bacterial pathogens across the MHS. Efforts such as this provide valuable context, relevant data, and actionable feedback on AST practices to public health and clinical decision-makers, which are vital to effectively support stewardship initiatives. Although there are other systems monitoring resistance patterns in non-military populations, it is important to have surveillance specifically targeted to military SMs, as they are a unique population with unique exposures. Actively monitoring enteric bacterial infections and AMR can be a valuable surveillance effort to support MTFs in identifying potential new AMR threats to the health and readiness of the force, particularly those serving in regions outside the United States.

## Supplementary Information

**Additional file 1: Supplementary Table 1.** Search terms used to query laboratory data.

**Additional file 2: Supplementary Table 2.** Frequency of target organism detections, stratified by Geographic Combatant Command, MHS, 2009-2019.

**Additional file 3: Supplementary Table 3.** Frequency of Stool and Non-stool by organism with susceptibility results, MHS, 2009-2019.

**Additional file 4: Supplementary Table 4.** Frequency of Specimens with Antibiotic Susceptibility Testing Results, by Genus and Antibiotic, MHS, 2009-2019 (*n* = 13,852).

**Additional file 5: Supplementary Table 5.** Susceptibility Patterns of Enteric Pathogens Across the MHS, 2009-2019.

**Additional file 6: Supplementary Table 6a.** Frequency of *Campylobacter* spp. Specimens with Antibiotic Susceptibility Results by Year, MHS, 2009-2019 (*n* = 182). **Supplementary Table 6b.** Frequency of STEC Specimens with Antibiotic Susceptibility Results by Year, MHS, 2009-2019 (*n* = 102).

## Data Availability

The data has been aggregated from non-publicly available datasets. The datasets generated and/or analyzed during the current study are not publicly available due to national security concerns. Please contact Gosia Nowak at gosia.nowak.civ@health.mil to request the dataset.

## Abbreviations

AD: Acute diarrhea
SM: Service members
AMR: Antimicrobial resistance
MHS: Military Health System
CONUS: Continental United States
OCONUS: Outside the Continental United States
HL7: Health Level 7
STEC: Shiga toxin-producing *Escherichia coli*
AST: Antibiotic susceptibility testing
CHCS: Composite Health Care System
MTF: Military treatment facility
MIC: Minimal inhibitory concentration
GCC: Geographic Combatant Command
NARMS: National Antimicrobial Resistance Monitoring System
NMCPHC: Navy and Marine Corps Public Health Center
KC: Kathleen Creppage
UC: Uzo Chukwuma
SB: Sara Bazaco
MJ: Myles Johnson
PG: Paul Graf
HA: Hayley Ashbaugh
KM: Kenji Matsumoto
UP: Upendra Bhattarai
NS: Nicholas Seliga
