# Supplementary material for: Antimicrobial resistance of enteric pathogens in the Military Health System, 2009 – 2019
Source: BMC Public Health. 2022 Dec 8;22:2300. doi: 10.1186/s12889-022-14466-1 (PMC9733093; doi:10.1186/s12889-022-14466-1)
Supplement: Supplementary file 1 — Additional file 1: Supplementary Table 1. Search terms used to query laboratory data. [file 12889_2022_14466_MOESM1_ESM.docx]

Supplementary Table 1. Search terms used to query laboratory data.

| Pathogen | Search terms |
| --- | --- |
| *Campylobacter* species | “Campylobacter species”, “Campy” |
| *Escherichia coli* | “0157”, “O157”, “Shiga”, “Stec”, “Enterohemorrhagic”, “Ehec”, “Verotoxin”, “Vtec”, “Verocytotoxin” |
| *Salmonella* species | “Salmonella species”, “Salmonella enterica”, “Salm” |
| *Shigella* species | “Shigella species”, “Shig”, “Flexneri”, “Dysenteriae”, “S. Boydii”, “S Boydii” |
