# Supplementary material for: Antimicrobial resistance of enteric pathogens in the Military Health System, 2009 – 2019
Source: BMC Public Health. 2022 Dec 8;22:2300. doi: 10.1186/s12889-022-14466-1 (PMC9733093; doi:10.1186/s12889-022-14466-1)
Supplement: Supplementary file 2 — Additional file 2: Supplementary Table 2. Frequency of target organism detections, stratified by Geographic Combatant Command, MHS, 2009-2019. [file 12889_2022_14466_MOESM2_ESM.docx]

| Supplementary Table 2: Frequency of target organism detections, stratified by Geographic Combatant Command, MHS, 2009-2019 | | | | | | | | | | | | |
| --- | --- | --- | --- | --- | --- | --- | --- | --- | --- | --- | --- | --- |
| **Geographic Combatant Command** | | | | | | | | | | | | |
|  | **CENTCOM** | | **EUCOM** | | **INDOCOM** | | **NORTHCOM** | | **SOUTHCOM** | | **TOTAL** | |
|  | Frequency (n) | Percentage of isolates (%) | Frequency (n) | Percentage of isolates (%) | Frequency (n) | Percentage of isolates (%) | Frequency (n) | Percentage of isolates (%) | Frequency (n) | Percentage of isolates (%) | Frequency (n) | Percentage of isolates (%) |
| **Bacterial Genus** |  |  |  |  |  |  |  |  |  |  |  |  |
| Shiga toxin-producing *E. coli* | 0 | 0% | 108 | 9% | 9 | 1% | 574 | 5% | 0 | 0% | 691 | 5% |
| *Campylobacter* | 0 | 0% | 757 | 63% | 364 | 50% | 3,805 | 32% | 1 | 9% | 4,927 | 36% |
| *Salmonella* | 32 | 100% | 301 | 25% | 335 | 46% | 6,078 | 51% | 9 | 82% | 6,755 | 49% |
| *Shigella* | 0 | 0% | 33 | 3% | 25 | 3% | 1,420 | 12% | 1 | 9% | 1,479 | 11% |
| Total | 32 | 0.2% | 1,199 | 9% | 733 | 5% | 11,877 | 86% | 11 | 0.1% | 13,852 | 100% |
| *Per cent is calculated as the number of isolates in a given category divided by the total number of isolates for that genus. | | | | | | | | | | | | |
| Data source: Health Level 7 (HL7)-formatted microbiology and chemistry databases. | | | | | | | | | | | | |
| Prepared by the EpiData Center, NMCPHC, Aug 2022. | | | | | | | | | | | | |
