# Supplementary material for: Antimicrobial resistance of enteric pathogens in the Military Health System, 2009 – 2019
Source: BMC Public Health. 2022 Dec 8;22:2300. doi: 10.1186/s12889-022-14466-1 (PMC9733093; doi:10.1186/s12889-022-14466-1)
Supplement: Supplementary file 3 — Additional file 3: Supplementary Table 3. Frequency of Stool and Non-stool by organism with susceptibility results, MHS, 2009-2019. [file 12889_2022_14466_MOESM3_ESM.docx]

| Supplementary Table 3: Frequency of Stool and Non-stool by organism with susceptibility results, MHS, 2009-2019 | | | | | | | | | | | |
| --- | --- | --- | --- | --- | --- | --- | --- | --- | --- | --- | --- |
|  | **Stool** | | | | **Non-Stool** | | | | **Number Detected** | **AST* (n)** | **AST (%^◆^)** |
|  | **CONUS** | | **OCONUS** | | **CONUS** | | **OCONUS** | |  |  |  |
|  | No. with AST (n) | % of those with AST (%)* | No. with AST (n) | % of those with AST (%)* | No. with AST (n) | % of those with AST (%)* | No. with AST (n) | % of those with AST (%)* |  |  |  |
| **Bacterial Genus** |  |  |  |  |  |  |  |  |  |  |  |
| Shiga toxin-producing *E. coli* | 44 | 43 | 8 | 8 | 37 | 36 | 13 | 13 | 691 | 102 | 15 |
| *Campylobacter* | 146 | 80 | 31 | 17 | 2 | 1 | 3 | 2 | 4,927 | 182 | 4 |
| *Salmonella* | 4,035 | 80 | 359 | 7 | 556 | 11 | 69 | 1 | 6,755 | 5,019 | 74 |
| *Shigella* | 1,045 | 86 | 33 | 3 | 130 | 11 | 13 | 1 | 1,479 | 1,221 | 83 |
| Total | 5,270 |  | 431 |  | 725 |  | 98 |  | 13,852 | 6,524 | 100% |
| *Antimicrobial susceptibility testing (AST). This column indicates the number of detected organisms undergoing AST. | | | | | | | | | | | |
| ^◆^Per cent is calculated as the number of isolates in a given category divided by the total number of isolates for that genus. | | | | | | | | | | | |
| Data source: Health Level 7 (HL7)-formatted microbiology and chemistry databases. | | | | | | | | | | | |
| Prepared by the EpiData Center, NMCPHC, on 18 Aug 2022. | | | | | | | | | | | |
