# Supplementary material for: Antimicrobial resistance of enteric pathogens in the Military Health System, 2009 – 2019
Source: BMC Public Health. 2022 Dec 8;22:2300. doi: 10.1186/s12889-022-14466-1 (PMC9733093; doi:10.1186/s12889-022-14466-1)
Supplement: Supplementary file 4 — Additional file 4: Supplementary Table 4. Frequency of Specimens with Antibiotic Susceptibility Testing Results, by Genus and Antibiotic, MHS, 2009-2019 (n = 13,852). [file 12889_2022_14466_MOESM4_ESM.docx]

| Supplementary Table 4: Frequency of Specimens with Antibiotic Susceptibility Testing Results, by Genus and Antibiotic, MHS, 2009-2019 (n=13,852) | | | | | | |  |
| --- | --- | --- | --- | --- | --- | --- | --- |
| **Antibiotic** | ***Campylobacter* spp. specimens with AST* (n)** | **STEC^◆^ specimens with AST (n)** | ***Salmonella* spp. specimens with AST (n)** | ***Shigella* spp. specimens with AST (n)** | **Total specimens with AST (n)** | **Total specimens with AST (%^◇^)** |  |
|  |  |  |  |  |  |  |  |
| Amikacin | 0 | 18 | 135 | 23 | 176 | 3 |  |
| Amoxicillin | 0 | 1 | 33 | 1 | 35 | 1 |  |
| Amoxicillin/Clavulanate | 9 | 66 | 314 | 79 | 468 | 7 |  |
| Ampicillin | 131 | 100 | 4,832 | 1,098 | 6,161 | 94 |  |
| Ampicillin/Sulbactam | 13 | 57 | 381 | 87 | 538 | 8 |  |
| Aztreonam | 4 | 24 | 203 | 64 | 295 | 5 |  |
| Carbenicillin | 0 | 0 | 8 | 0 | 8 | 0.1 |  |
| Cefaclor | 0 | 0 | 17 | 0 | 17 | 0.3 |  |
| Cefamandole | 0 | 0 | 3 | 0 | 3 | 0.05 |  |
| Cefazolin | 15 | 83 | 425 | 97 | 620 | 10 |  |
| Cefepime | 19 | 53 | 469 | 96 | 637 | 10 |  |
| Cefixime | 0 | 0 | 8 | 0 | 8 | 0.1 |  |
| Cefmetazole | 0 | 0 | 12 | 0 | 12 | 0.2 |  |
| Cefoperazone | 0 | 0 | 4 | 0 | 4 | 0.06 |  |
| Cefotaxime | 6 | 38 | 216 | 35 | 295 | 5 |  |
| Cefotetan | 1 | 8 | 26 | 7 | 42 | 1 |  |
| Cefoxitin | 6 | 21 | 159 | 12 | 198 | 3 |  |
| Cefpodoxime | 0 | 1 | 4 | 1 | 6 | 0.09 |  |
| Ceftazidime | 21 | 53 | 660 | 119 | 853 | 13 |  |
| Ceftizoxime | 0 | 0 | 2 | 0 | 2 | 0.03 |  |
| Ceftriaxone | 23 | 75 | 999 | 176 | 1,273 | 20 |  |
| Cefuroxime | 4 | 48 | 92 | 28 | 172 | 3 |  |
| Cefuroxime axetil | 0 | 1 | 15 | 0 | 16 | 0.2 |  |
| Cephalothin | 2 | 20 | 20 | 7 | 49 | 1 |  |
| Ciprofloxacin | 126 | 93 | 3,768 | 991 | 4,978 | 76 |  |
| Chloramphenicol | 0 | 0 | 13 | 1 | 14 | 0.2 |  |
| Clindamycin | 7 | 0 | 27 | 5 | 39 | 1 |  |
| Doripenem | 0 | 0 | 21 | 10 | 31 | 0.5 |  |
| Doxycyline | 1 | 0 | 29 | 1 | 31 | 0.5 |  |
| Ertapenem | 2 | 15 | 133 | 28 | 178 | 3 |  |
| Erythromycin | 33 | 0 | 30 | 4 | 67 | 1 |  |
| Gatifloxacin | 0 | 7 | 23 | 7 | 37 | 1 |  |
| Gemifloxacin | 0 | 5 | 4 | 0 | 9 | 0.1 |  |
| Gentamicin | 39 | 92 | 513 | 115 | 759 | 12 |  |
| Imipenem | 21 | 47 | 427 | 80 | 575 | 9 |  |
| Levofloxacin | 49 | 73 | 2,266 | 632 | 3,020 | 46 |  |
| Meropenem | 7 | 14 | 303 | 43 | 367 | 6 |  |
| Mezlocillin | 0 | 0 | 3 | 0 | 3 | 0.05 |  |
| Minocycline | 0 | 0 | 23 | 0 | 23 | 0.4 |  |
| Moxalactam | 0 | 0 | 4 | 0 | 4 | 0.06 |  |
| Moxifloxacin | 1 | 0 | 64 | 3 | 68 | 1 |  |
| Nalidixic Acid | 2 | 0 | 8 | 0 | 10 | 0.2 |  |
| Nitrofurantoin | 1 | 47 | 253 | 88 | 389 | 6 |  |
| Norfloxacin | 0 | 2 | 36 | 1 | 39 | 1 |  |
| Ofloxacin | 0 | 0 | 2 | 1 | 3 | 0.05 |  |
| Penicillin | 1 | 0 | 7 | 1 | 9 | 0.1 |  |
| Piperacillin | 3 | 17 | 247 | 38 | 305 | 5 |  |
| Piperacillin/Tazobactam | 11 | 51 | 361 | 65 | 488 | 7 |  |
| Tetracycline | 15 | 46 | 164 | 38 | 263 | 4 |  |
| Ticarcillin | 0 | 0 | 10 | 0 | 10 | 0.2 |  |
| Ticarcillin/Clavulanate | 2 | 19 | 51 | 13 | 85 | 1 |  |
| Tigecycline | 0 | 2 | 55 | 8 | 65 | 1 |  |
| Tobramycin | 13 | 62 | 233 | 64 | 372 | 6 |  |
| Trimethoprim | 0 | 3 | 8 | 5 | 16 | 0.2 |  |
| Trimethoprim/ Sulfamethoxazole | 129 | 97 | 4,813 | 1,165 | 6,204 | 95 |  |
| *AST = Antibiotic Susceptibility Testing. A specimen could have more than one AST result for more than one antibiotic. | | | | | | |  |
| ^◆^STEC = Shiga toxin-producing *E. coli* | | | | | | |  |
| ^◇^Percentages in the total column are based off the total number of specimens undergoing AST for a given antibiotic. | | | | | | |  |
| Data source: HL7-formatted laboratory CHCS data. | | | | | | |  |
