# Supplementary material for: Antimicrobial resistance of enteric pathogens in the Military Health System, 2009 – 2019
Source: BMC Public Health. 2022 Dec 8;22:2300. doi: 10.1186/s12889-022-14466-1 (PMC9733093; doi:10.1186/s12889-022-14466-1)
Supplement: Supplementary file 5 — Additional file 5: Supplementary Table 5. Susceptibility Patterns of Enteric Pathogens Across the MHS, 2009-2019. [file 12889_2022_14466_MOESM5_ESM.docx]

| Supplementary Table 5. Susceptibility Patterns of Enteric Pathogens Across the MHS, 2009-2019 | | | | | | | | | | | | | | | | | | | |
| --- | --- | --- | --- | --- | --- | --- | --- | --- | --- | --- | --- | --- | --- | --- | --- | --- | --- | --- | --- |
|  | | 2009 | | | | | | 2010 | | | | | | 2011 | | | | | |
| Antibiotics | | CONUS | | | OCONUS | | | CONUS | | | OCONUS | | | CONUS | | | OCONUS | | |
|  |  | n | S* (n) | S (%①) | n | S (n) | S (%) | n | S (n) | S (%) | n | S (n) | S (%) | n | S (n) | S (%) | n | S (n) | S (%) |
| ***Salmonella* species** | | | | | | | | | | | | | | | | | | | |
| AMPICILLIN | | 469 | 439 | 93.6% | 46 | 35 | 76.1%^◊^ | 496 | 468 | 94.4% | 51 | 43 | 84.3% | 500 | 466 | 93.2% | 41 | 33 | 80.5% |
| CEFTRIAXONE | | 115 | 115 | 100.0% | 14^ꝉ^ | 13 | -- | 104 | 104 | 100.0% | 23^ꝉ^ | 23 | -- | 102 | 102 | 100.0% | 21^ꝉ^ | 19 | -- |
| CIPROFLOXACIN | | 391 | 391 | 100.0% | 39 | 39 | 100.0% | 401 | 401 | 100.0% | 46 | 45 | 97.8% | 412 | 411 | 99.8% | 48 | 46 | 95.8% |
| IMIPENEM | | 37 | 37 | 100.0% | 13^ꝉ^ | 13 | -- | 57 | 57 | 100.0% | 18^ꝉ^ | 18 | -- | 51 | 51 | 100.0% | 25^ꝉ^ | 25 | -- |
| LEVOFLOXACIN | | 242 | 240 | 99.2% | 29^ꝉ^ | 29 | -- | 256 | 256 | 100.0% | 34 | 33 | 97.1% | 233 | 232 | 99.6% | 28 | 27 | 96.4% |
| MEROPENEM | | 21^ꝉ^ | 21 | -- | 13^ꝉ^ | 13 | -- | 35 | 35 | 100.0% | 15^ꝉ^ | 15 | -- | 37 | 37 | 100.0% | 12^ꝉ^ | 12 | -- |
| TRIMETHOPRIM- SULFAMETHOXAZOLE | | 469 | 461 | 98.3% | 41 | 38 | 92.7% | 473 | 465 | 98.3% | 53 | 49 | 92.5% | 479 | 474 | 99.0% | 47 | 44 | 93.6% |
| ***Shigella* species** | | | | | | | | | | | | | | | | | | | |
| AMPICILLIN | | 108 | 68 | 63.0%^◊^ | 4^ꝉ^ | 4 | -- | 154 | 101 | 65.6%^◊^ | 3^ꝉ^ | 1 | -- | 76 | 49 | 64.5% ^◊^ | 7^ꝉ^ | 5 | -- |
| CEFTRIAXONE | | 15^ꝉ^ | 15 | -- | 1^ꝉ^ | 1 | -- | 23^ꝉ^ | 23 | -- | 0^ꝉ^ | 0 | -- | 17^ꝉ^ | 16 | -- | 1^ꝉ^ | 1 | -- |
| CIPROFLOXACIN | | 100 | 99 | 99.0% | 4^ꝉ^ | 4 | -- | 134 | 134 | 100.0% | 3^ꝉ^ | 2 | -- | 56 | 53 | 94.6% | 9^ꝉ^ | 7 | -- |
| LEVOFLOXACIN | | 75 | 75 | 100.0% | 1^ꝉ^ | 1 | -- | 73 | 72 | 98.6% | 0^ꝉ^ | 0 | -- | 32 | 32 | 100.0% | 3^ꝉ^ | 3 | -- |
| TRIMETHOPRIM- SULFAMETHOXAZOLE | | 120 | 60 | 50.0%^◊^ | 4^ꝉ^ | 2 | -- | 150 | 58 | 38.7%^◊^ | 3^ꝉ^ | 1 | -- | 73 | 28 | 38.4% ^◊^ | 8^ꝉ^ | 1 | -- |
|  |  |  |  |  |  |  |  |  |  |  |  |  |  |  |  |  |  |  |  |
|  |  | 2012 | | | | | | 2013 | | | | | | 2014 | | | | | |
| Antibiotics | | CONUS | | | OCONUS | | | CONUS | | | OCONUS | | | CONUS | | | OCONUS | | |
|  |  | n | S (n) | S (%) | n | S (n) | S (%) | n | S (n) | S (%) | n | S (n) | S (%) | n | S (n) | S (%) | n | S (n) | S (%) |
| ***Salmonella* species** | | | | | | | | | | | | | | | | | | | |
| AMPICILLIN | | 433 | 401 | 92.6% | 28^ꝉ^ | 22 | -- | 386 | 362 | 93.8% | 20^ꝉ^ | 17 | -- | 445 | 416 | 93.5% | 33 | 28 | 84.8% |
| CEFTRIAXONE | | 83 | 83 | 100.0% | 14^ꝉ^ | 14 | -- | 79 | 78 | 98.7% | 12^ꝉ^ | 12 | -- | 76 | 76 | 100.0% | 8^ꝉ^ | 8 | -- |
| CIPROFLOXACIN | | 371 | 369 | 99.5% | 38 | 34 | 89.5% | 324 | 321 | 99.1% | 22^ꝉ^ | 22 | -- | 348 | 343 | 98.6% | 30 | 30 | 100.0% |
| IMIPENEM | | 49 | 48 | 98.0% | 17^ꝉ^ | 17 | -- | 28ꝉ | 28 | -- | 11^ꝉ^ | 11 | -- | 17^ꝉ^ | 17 | -- | 6^ꝉ^ | 6 | -- |
| LEVOFLOXACIN | | 216 | 216 | 100.0% | 20^ꝉ^ | 18 | -- | 166 | 165 | 99.4% | 15^ꝉ^ | 15 | -- | 179 | 179 | 100.0% | 19^ꝉ^ | 19 | -- |
| MEROPENEM | | 31 | 31 | 100.0% | 14^ꝉ^ | 14 | -- | 9^ꝉ^ | 9 | -- | 13^ꝉ^ | 13 | -- | 15^ꝉ^ | 15 | -- | 7^ꝉ^ | 7 | -- |
| TRIMETHOPRIM- SULFAMETHOXAZOLE | | 416 | 408 | 98.1% | 38 | 35 | 92.1% | 393 | 391 | 99.5% | 19^ꝉ^ | 19 | -- | 455 | 446 | 98.0% | 29 | 26 | 89.7% |
| ***Shigella* species** | | | | | | | | | | | | | | | | | | | |
| AMPICILLIN | | 81 | 62 | 76.5% ^◊^ | 2^ꝉ^ | 1 | -- | 53 | 27 | 51.0% ^◊^ | 4^ꝉ^ | 2 | -- | 135 | 91 | 67.4%^◊^ | 2^ꝉ^ | 1 | -- |
| CEFTRIAXONE | | 20^ꝉ^ | 20 | -- | 1^ꝉ^ | 1 | -- | 8^ꝉ^ | 8 | -- | 0^ꝉ^ | 0 | -- | 21^ꝉ^ | 21 | -- | 1^ꝉ^ | 1 | -- |
| CIPROFLOXACIN | | 78 | 77 | 98.7% | 2^ꝉ^ | 2 | -- | 41 | 40 | 97.6% | 4^ꝉ^ | 3 | -- | 145 | 140 | 96.6% | 2^ꝉ^ | 1 | -- |
| LEVOFLOXACIN | | 62 | 60 | 96.8% | 1^ꝉ^ | 1 | -- | 37 | 37 | 100.0% | 0^ꝉ^ | 0 | -- | 83 | 80 | 96.4% | 1^ꝉ^ | 1 | -- |
| TRIMETHOPRIM- SULFAMETHOXAZOLE | | 82 | 50 | 61.0% ^◊^ | 2^ꝉ^ | 1 | -- | 52 | 28 | 53.8% ^◊^ | 4^ꝉ^ | 1 | -- | 161 | 64 | 39.7%^◊^ | 2`^ꝉ^ | 1 | -- |
|  |  |  |  |  |  |  |  |  |  |  |  |  |  |  |  |  |  |  |  |
|  |  | 2015 | | | | | | 2016 | | | | | | 2017 | | | | | |
| Antibiotics | | CONUS | | | OCONUS | | | CONUS | | | OCONUS | | | CONUS | | | OCONUS | | |
|  |  | n | S (n) | S (%) | n | S (n) | S (%) | n | S (n) | S (%) | n | S (n) | S (%) | n | S (n) | S (%) | n | S (n) | S (%) |
| ***Salmonella* species** | | | | | | | | | | | | | | | | | | | |
| AMPICILLIN | | 430 | 404 | 94.0% | 31 | 26 | 83.9% | 457 | 424 | 92.8% | 38 | 31 | 81.6% | 283 | 257 | 90.8% | 34 | 28^ꝉ^ | -- |
| CEFTRIAXONE | | 92 | 90 | 97.8% | 6^ꝉ^ | 6 | -- | 55 | 51 | 92.7% | 12^ꝉ^ | 12 | -- | 41 | 40 | 97.6% | 14^ꝉ^ | 14 | -- |
| CIPROFLOXACIN | | 290 | 274 | 94.5% | 28^ꝉ^ | 26 | -- | 307 | 304 | 99.0% | 22^ꝉ^ | 22 | -- | 194 | 192 | 99.0% | 32 | 31 | 96.9% |
| IMIPENEM | | 32 | 32 | 100.0% | 4^ꝉ^ | 4 | -- | 14^ꝉ^ | 14 | -- | 9^ꝉ^ | 9 | -- | 8^ꝉ^ | 8 | -- | 10^ꝉ^ | 10 | -- |
| LEVOFLOXACIN | | 179 | 177 | 98.9% | 14^ꝉ^ | 14 | -- | 220 | 218 | 99.1% | 23^ꝉ^ | 23 | -- | 115 | 115 | 100.0% | 21^ꝉ^ | 21 | -- |
| MEROPENEM | | 19^ꝉ^ | 19 | -- | 7^ꝉ^ | 7 | -- | 5^ꝉ^ | 5 | -- | 6^ꝉ^ | 6 | -- | 4^ꝉ^ | 4 | -- | 9^ꝉ^ | 9 | -- |
| TRIMETHOPRIM- SULFAMETHOXAZOLE | | 431 | 425 | 98.6% | 30 | 29 | 96.7% | 455 | 445 | 97.8% | 36 | 32 | 88.9% | 294 | 287 | 97.6% | 33 | 29 | 87.9% |
| ***Shigella* species** | | | | | | | | | | | | | | | | | | | |
| AMPICILLIN | | 210 | 178 | 84.8% | 4^ꝉ^ | 4 | -- | 101 | 61 | 60.4% ^◊^ | 7^ꝉ^ | 4 | -- | 53 | 23 | 43.4% ^◊^ | 4^ꝉ^ | 2 | -- |
| CEFTRIAXONE | | 16^ꝉ^ | 16 | -- | 1^ꝉ^ | 1 | -- | 16^ꝉ^ | 16 | -- | 1^ꝉ^ | 1 | -- | 9^ꝉ^ | 9 | -- | 2^ꝉ^ | 1 | -- |
| CIPROFLOXACIN | | 177 | 173 | 97.7% | 3^ꝉ^ | 3 | -- | 100 | 92 | 92.0% | 4^ꝉ^ | 4 | -- | 51 | 45 | 88.2% | 3^ꝉ^ | 3 | -- |
| LEVOFLOXACIN | | 82 | 78 | 95.1% | 2^ꝉ^ | 2 | -- | 70 | 68 | 97.1% | 4^ꝉ^ | 3 | -- | 46 | 41 | 89.1% | 5^ꝉ^ | 4 | -- |
| TRIMETHOPRIM- SULFAMETHOXAZOLE | | 277 | 121 | 43.7% ^◊^ | 4^ꝉ^ | 1 | -- | 112 | 58 | 51.8% ^◊^ | 6^ꝉ^ | 0 | -- | 56 | 19 | 33.9% ^◊^ | 4^ꝉ^ | 2 | -- |
|  |  |  |  |  |  |  |  |  |  |  |  |  |  |  |  |  |  |  |  |
|  |  | 2018 | | | | | | 2019 | | | | | |  |  |  |  |  |  |
| Antibiotics | | CONUS | | | OCONUS | | | CONUS | | | OCONUS | | |  |  |  |  |  |  |
|  |  | n | S (n) | S (%) | n | S (n) | S (%) | n | S (n) | S (%) | n | S (n) | S (%) |  |  |  |  |  |  |
| ***Salmonella* species** | | | | | | | | | | | | | |  |  |  |  |  |  |
| AMPICILLIN | | 312 | 286 | 91.7% | 23^ꝉ^ | 20 | -- | 241 | 225 | 93.4% | 35 | 30 | 85.7% |  |  |  |  |  |  |
| CEFTRIAXONE | | 57 | 53 | 93.0% | 8^ꝉ^ | 8 | -- | 52 | 51 | 98.1% | 11^ꝉ^ | 11 | -- |  |  |  |  |  |  |
| CIPROFLOXACIN | | 210 | 207 | 98.6% | 22^ꝉ^ | 21 | -- | 158 | 157 | 99.4% | 35 | 29^ꝉ^ | -- |  |  |  |  |  |  |
| IMIPENEM | | 8^ꝉ^ | 8 | -- | 5^ꝉ^ | 5 | -- | 4^ꝉ^ | 4 | -- | 4^ꝉ^ | 4 | -- |  |  |  |  |  |  |
| LEVOFLOXACIN | | 131 | 131 | 100.0% | 12^ꝉ^ | 12 | -- | 102 | 100 | 98.0% | 12^ꝉ^ | 11 | -- |  |  |  |  |  |  |
| MEROPENEM | | 13^ꝉ^ | 12 | -- | 2^ꝉ^ | 2 | -- | 13^ꝉ^ | 13 | -- | 3^ꝉ^ | 3 | -- |  |  |  |  |  |  |
| TRIMETHOPRIM- SULFAMETHOXAZOLE | | 323 | 313 | 96.9% | 18^ꝉ^ | 18 | -- | 246 | 240 | 97.6% | 35 | 33 | 94.3% |  |  |  |  |  |  |
| ***Shigella* species** | | | | | | | | | | | | | |  |  |  |  |  |  |
| AMPICILLIN | | 27^ꝉ^ | 22 | -- | 3^ꝉ^ | 2 | -- | 57 | 41 | 71.9% ^◊^ | 3^ꝉ^ | 2 | -- |  |  |  |  |  |  |
| CEFTRIAXONE | | 7^ꝉ^ | 6 | -- | 1^ꝉ^ | 1 | -- | 14^ꝉ^ | 14 | -- | 1^ꝉ^ | 1 | -- |  |  |  |  |  |  |
| CIPROFLOXACIN | | 23^ꝉ^ | 15 | -- | 3^ꝉ^ | 3 | -- | 46 | 41 | 89.1% | 3^ꝉ^ | 2 | -- |  |  |  |  |  |  |
| LEVOFLOXACIN | | 14^ꝉ^ | 11 | -- | 2^ꝉ^ | 2 | -- | 37 | 36 | 97.3% | 2^ꝉ^ | 1 | -- |  |  |  |  |  |  |
| TRIMETHOPRIM- SULFAMETHOXAZOLE | | 31 | 9 | 29.0%^◊^ | 3^ꝉ^ | 2 | -- | 58 | 22 | 37.9% ^◊^ | 3^ꝉ^ | 0 | -- |  |  |  |  |  |  |
|  |  |  |  |  |  |  |  |  |  |  |  |  |  |  |  |  |  |  |  |
| * | S = Susceptible |  |  |  |  |  |  |  |  |  |  |  |  |  |  |  |  |  |  |
| ① | Indicates the per cent of all specimens for a particular genus susceptible to a given antibiotic for a given year | | | | | | | |  |  |  |  |  |  |  |  |  |  |  |
| -- | The percent susceptibility was not calculated due to the minimum specimen threshold (n=30) not being met | | | | | | |  |  |  |  |  |  |  |  |  |  |  |  |
| ꝉ | Minimum specimen threshold (n=30) was not met | | | |  |  |  |  |  |  |  |  |  |  |  |  |  |  |  |
| ◊ | Percent susceptibility for all specimens tested against that antibiotic was below 80% | | | | | |  |  |  |  |  |  |  |  |  |  |  |  |  |
