# Supplementary material for: Antimicrobial resistance of enteric pathogens in the Military Health System, 2009 – 2019
Source: BMC Public Health. 2022 Dec 8;22:2300. doi: 10.1186/s12889-022-14466-1 (PMC9733093; doi:10.1186/s12889-022-14466-1)
Supplement: Supplementary file 6 — Additional file 6: Supplementary Table 6a. Frequency of Campylobacter spp. Specimens with Antibiotic Susceptibility Results by Year, MHS, 2009-2019 (n = 182). Supplementary Table 6b. Frequency of STEC Specimens with Antibiotic Susceptibility Results by Year, MHS, 2009-2019 (n = 102). [file 12889_2022_14466_MOESM6_ESM.docx]

Supplementary Table 6a. Frequency of *Campylobacter* spp. Specimens with Antibiotic Susceptibility Results by Year, MHS, 2009-2019 (n=182)

| **Antibiotics** | **2009** | **2010** | **2011** | **2012** | **2013** | **2014** | **2015** | **2016** | **2017** | **2018** | **2019** | **Total** |
| --- | --- | --- | --- | --- | --- | --- | --- | --- | --- | --- | --- | --- |
|  | n | n | n | n | N | n | n | n | n | n | n | n |
| Amikacin | 0 | 0 | 0 | 0 | 0 | 0 | 0 | 0 | 0 | 0 | 0 | 0 |
| Ampicillin | 3 | 3 | 7 | 11 | 5 | 9 | 10 | 17 | 26 | 21 | 19 | 131 |
| Ampicillin /Sulbactam | 1 | 0 | 1 | 2 | 2 | 1 | 0 | 0 | 1 | 1 | 4 | 13 |
| Cefepime | 1 | 0 | 1 | 2 | 1 | 1 | 3 | 0 | 4 | 2 | 4 | 19 |
| Cefotaxime | 0 | 0 | 1 | 0 | 0 | 1 | 1 | 0 | 2 | 0 | 1 | 6 |
| Cefoxitin | 0 | 0 | 1 | 0 | 0 | 1 | 1 | 0 | 1 | 0 | 2 | 6 |
| Cephalothin | 0 | 0 | 1 | 1 | 0 | 0 | 0 | 0 | 0 | 0 | 0 | 2 |
| Ceftazidime | 0 | 0 | 3 | 1 | 1 | 1 | 3 | 2 | 3 | 3 | 4 | 21 |
| Ceftriaxone | 1 | 0 | 2 | 2 | 2 | 1 | 3 | 1 | 5 | 3 | 3 | 23 |
| Chloramphenicol | 0 | 0 | 0 | 0 | 0 | 0 | 0 | 0 | 0 | 0 | 0 | 0 |
| Ciprofloxacin | 6 | 7 | 11 | 12 | 4 | 8 | 11 | 15 | 21 | 16 | 15 | 126 |
| Clindamycin | 0 | 0 | 0 | 0 | 0 | 2 | 2 | 1 | 1 | 1 | 0 | 7 |
| Erythromycin | 6 | 12 | 4 | 0 | 0 | 3 | 4 | 1 | 1 | 1 | 1 | 33 |
| Gentamicin | 2 | 1 | 3 | 3 | 3 | 2 | 6 | 2 | 7 | 4 | 6 | 39 |
| Imipenem | 0 | 0 | 3 | 0 | 0 | 1 | 2 | 1 | 5 | 5 | 4 | 21 |
| Levofloxacin | 1 | 0 | 3 | 4 | 2 | 3 | 6 | 8 | 7 | 6 | 9 | 49 |
| Meropenem | 0 | 0 | 1 | 0 | 0 | 1 | 1 | 1 | 2 | 0 | 1 | 7 |
| Minocycline | 0 | 0 | 0 | 0 | 0 | 0 | 0 | 0 | 0 | 0 | 0 | 0 |
| Penicillin | 0 | 0 | 0 | 0 | 0 | 1 | 0 | 0 | 0 | 0 | 0 | 1 |
| Piperacillin | 0 | 0 | 0 | 0 | 0 | 0 | 0 | 0 | 0 | 0 | 3 | 3 |
| Tetracycline | 0 | 0 | 0 | 1 | 1 | 1 | 2 | 2 | 2 | 3 | 3 | 15 |
| Ticarcillin | 0 | 0 | 0 | 0 | 0 | 0 | 0 | 0 | 0 | 0 | 0 | 0 |
| Ticarcillin /Clavulanate | 0 | 0 | 1 | 0 | 0 | 1 | 0 | 0 | 0 | 0 | 0 | 2 |
| Tigecycline | 0 | 0 | 0 | 0 | 0 | 0 | 0 | 0 | 0 | 0 | 0 | 0 |
| Tobramycin | 0 | 0 | 3 | 1 | 1 | 1 | 1 | 0 | 3 | 1 | 2 | 13 |
| Trimethoprim /Sulfamethoxazole | 2 | 2 | 6 | 12 | 5 | 10 | 13 | 15 | 25 | 19 | 20 | 129 |

Data source: HL7-formatted laboratory CHCS data.

Supplementary Table 6b. Frequency of STEC Specimens with Antibiotic Susceptibility Results by Year, MHS, 2009-2019 (n=102)

| Antibiotics | 2009 | 2010 | 2011 | 2012 | 2013 | 2014 | 2015 | 2016 | 2017 | 2018 | 2019 | Total |
| --- | --- | --- | --- | --- | --- | --- | --- | --- | --- | --- | --- | --- |
|  | n | n | n | n | n | n | n | n | n | n | n | n |
| Amikacin | 2 | 3 | 2 | 1 | 5 | 0 | 2 | 1 | 0 | 2 | 0 | 18 |
| Amoxicillin | 1 | 0 | 0 | 0 | 0 | 0 | 0 | 0 | 0 | 0 | 0 | 1 |
| Amoxicillin /Clavulanate | 14 | 8 | 3 | 3 | 8 | 6 | 5 | 9 | 3 | 6 | 1 | 66 |
| Ampicillin | 17 | 15 | 8 | 6 | 11 | 8 | 10 | 12 | 5 | 7 | 1 | 100 |
| Ampicillin /Sulbactam | 4 | 7 | 4 | 2 | 11 | 4 | 6 | 7 | 4 | 7 | 1 | 57 |
| Aztreonam | 4 | 3 | 0 | 0 | 7 | 1 | 3 | 2 | 1 | 3 | 0 | 24 |
| Cefazolin | 15 | 11 | 7 | 4 | 11 | 5 | 7 | 11 | 4 | 7 | 1 | 83 |
| Cefepime | 3 | 5 | 2 | 2 | 10 | 7 | 7 | 8 | 4 | 4 | 1 | 53 |
| Cefotaxime | 7 | 5 | 4 | 2 | 8 | 4 | 4 | 1 | 1 | 2 | 0 | 38 |
| Cefotetan | 3 | 1 | 2 | 2 | 0 | 0 | 0 | 0 | 0 | 0 | 0 | 8 |
| Cefoxitin | 7 | 3 | 1 | 1 | 0 | 0 | 1 | 3 | 1 | 4 | 0 | 21 |
| Ceftazidime | 10 | 6 | 3 | 3 | 8 | 1 | 5 | 8 | 5 | 4 | 0 | 53 |
| Ceftriaxone | 8 | 9 | 5 | 4 | 10 | 7 | 9 | 11 | 5 | 6 | 1 | 75 |
| Cefuroxime | 5 | 6 | 5 | 3 | 11 | 3 | 4 | 4 | 3 | 4 | 0 | 48 |
| Cefuroxime axetil | 0 | 0 | 0 | 0 | 0 | 0 | 0 | 0 | 1 | 0 | 0 | 1 |
| Cephalothin | 4 | 7 | 0 | 0 | 3 | 3 | 3 | 0 | 0 | 0 | 0 | 20 |
| Ciprofloxacin | 15 | 15 | 7 | 5 | 11 | 8 | 8 | 11 | 6 | 7 | 0 | 93 |
| Ertapenem | 2 | 2 | 0 | 0 | 3 | 0 | 4 | 3 | 0 | 0 | 1 | 15 |
| Gatifloxacin | 2 | 1 | 0 | 0 | 2 | 0 | 1 | 1 | 0 | 0 | 0 | 7 |
| Gemifloxacin | 1 | 1 | 0 | 0 | 3 | 0 | 0 | 0 | 0 | 0 | 0 | 5 |
| Gentamicin | 14 | 13 | 8 | 5 | 11 | 7 | 9 | 11 | 6 | 7 | 1 | 92 |
| Imipenem | 7 | 5 | 3 | 3 | 8 | 1 | 3 | 8 | 3 | 5 | 1 | 47 |
| Levofloxacin | 7 | 9 | 6 | 3 | 8 | 8 | 7 | 11 | 5 | 8 | 1 | 73 |
| Meropenem | 2 | 1 | 1 | 3 | 0 | 0 | 2 | 2 | 1 | 1 | 1 | 14 |
| Nitrofurantoin | 7 | 9 | 6 | 1 | 3 | 7 | 6 | 2 | 3 | 3 | 0 | 47 |
| Norfloxacin | 0 | 1 | 0 | 0 | 0 | 0 | 0 | 1 | 0 | 0 | 0 | 2 |
| Piperacillin | 3 | 1 | 0 | 0 | 4 | 4 | 3 | 1 | 1 | 0 | 0 | 17 |
| Piperacillin /Tazobactam | 5 | 5 | 3 | 2 | 10 | 4 | 4 | 6 | 4 | 7 | 1 | 51 |
| Tetracycline | 12 | 8 | 1 | 0 | 7 | 4 | 3 | 4 | 2 | 5 | 0 | 46 |
| Ticarcillin /Clavulanate | 5 | 1 | 0 | 0 | 9 | 1 | 3 | 0 | 0 | 0 | 0 | 19 |
| Tigecycline | 0 | 1 | 0 | 0 | 0 | 0 | 0 | 0 | 0 | 1 | 0 | 2 |
| Tobramycin | 6 | 10 | 5 | 2 | 11 | 8 | 5 | 6 | 3 | 5 | 1 | 62 |
| Trimethoprim | 0 | 1 | 0 | 0 | 2 | 0 | 0 | 0 | 0 | 0 | 0 | 3 |
| Trimethoprim /Sulfamethoxazole | 15 | 15 | 7 | 6 | 11 | 8 | 9 | 12 | 6 | 8 | 0 | 97 |

STEC = Shiga toxin-producing *E. Coli*

Data source: HL7-formatted laboratory CHCS data.

Updated 17Aug22
